# Modulation of Methamphetamine-Related Attention Bias by Intermittent Theta-Burst Stimulation on Left Dorsolateral Prefrontal Cortex

**DOI:** 10.3389/fcell.2021.667476

**Published:** 2021-08-03

**Authors:** Tianzhen Chen, Hang Su, Lihui Wang, Xiaotong Li, Qianying Wu, Na Zhong, Jiang Du, Yiran Meng, Chunmei Duan, Congbin Zhang, Wen Shi, Ding Xu, Weidong Song, Min Zhao, Haifeng Jiang

**Affiliations:** ^1^Shanghai Mental Health Center, Shanghai Jiao Tong University School of Medicine, Shanghai, China; ^2^Institute of Psychology and Behavioral Science, Shanghai Jiao Tong University, Shanghai, China; ^3^Yunnan Institute on Drug Dependence, Kunming, China; ^4^Shanghai Female Compulsory Rehabilitation Center, Shanghai, China; ^5^Shanghai Drug Rehabilitation Administration Bureau, Shanghai, China; ^6^Shanghai Key Laboratory of Psychotic Disorders, Shanghai, China; ^7^Center for Excellence in Brain Science and Intelligence Technology, Chinese Academy of Sciences, Shanghai, China

**Keywords:** attention bias, dorsolateral prefrontal cortex, electroencephalogram, event-related potential, methamphetamine, transcranial magnetic stimulation

## Abstract

**Background:**

Previous studies have identified the treatment effect of repetitive transcranial magnetic stimulation (rTMS) on cravings of patients with methamphetamine use disorder (MUD). However, the mechanism underlying the treatment effect remains largely unknown. A potential candidate mechanism could be that rTMS over the dorsolateral prefrontal cortex (DLPFC) modulates the attention bias to methamphetamine-related cues. The purpose of this study is therefore to determine the modulation of rTMS on methamphetamine-related attention bias and the corresponding electrophysiological changes.

**Methods:**

Forty-nine patients with severe MUD were included for analysis. The subjects were randomized to receive the active intermittent theta-burst stimulation (iTBS) or sham iTBS targeting DLPFC for 20 sessions. Participants performed the Addiction Stroop Task before and after the treatment while being recorded by a 64-channel electroencephalogram. Baseline characteristics were collected through the Addiction Severity Index.

**Results:**

Post-treatment evaluations showed a reduced error rate in discriminating the color of methamphetamine words in the active iTBS group compared with the sham iTBS group. Following rTMS treatment, we found the significant time-by-group effect for the N1 amplitude (methamphetamine words > neutral words) and P3 latency (methamphetamine words > neutral words). The change of N1 amplitude was positively correlated with cravings in the active group. Moreover, reduced power of neural oscillation in the beta band, manifesting at frontal central areas, was also found in the active group.

**Conclusion:**

This study suggests that attention bias and the beta oscillation during the attentional processing of methamphetamine words in patients with MUD could be modulated by iTBS applied to left DLPFC.

## Introduction

Repetitive drug use and the formation of addiction are generally believed to be related to the responses to substance-related cues (Courtney et al., 2016). Several studies have demonstrated that patients with substance use disorder were prone to react with increasing subjective cravings and physiological arousal when exposed to substance-related stimuli (Carter and Tiffany, 1999; Norberg et al., 2016; Tan et al., 2019). Ryan suggested that attention bias reflects the deviation of patients’ cognitive processing of substance-related stimuli (Ryan, 2002). According to the integrated model proposed by Franken (2003), attention bias is the result of classical conditioning. This model suggests that the conditioned substance-related cue stimuli induce a dopaminergic response. These stimuli are then taken as “salient” and attract the attention of patients. The substance-related attention bias may also closely link to patients’ long-term drug use behaviors and outcomes (Field et al., 2014). As an important feature involving the development of substance dependence, stimulus-related attention bias has become one of the potential targets of various addiction interventions (den Uyl et al., 2018; Zhang M. W. et al., 2018; Zhang M. et al., 2019).

Repetitive transcranial magnetic stimulation (rTMS) could initiate cortical plasticity changes through brain intervention (Pell et al., 2011). The dorsolateral prefrontal cortex (DLPFC) has been recently identified as the most promising intervention site to capture tailored therapy effects in substance dependence (Zhang J. J. Q. et al., 2019). Previous MRI studies on substance-dependent patients [e.g., alcohol, methamphetamine (MA), cocaine, opiate, and nicotine] have found that the most reported regions that are related to cue reactivity were DLPFC, orbitofrontal cortex, amygdala, and anterior cingulate cortex, the activation of which were accompanied with a strong desire to reuse substance (Jasinska et al., 2014; Grodin et al., 2019). This may partially explain the underlying brain mechanism of rTMS intervention in DLPFC to reduce the cravings of substance-dependent patients by modulating the plasticity of DLPFC (Hayashi et al., 2013; Grodin et al., 2019), and leading to changes in psychological states and behaviors. However, there is still much to be investigated on the relationship between rTMS interventions in DLPFC and cue reactivity. Both attention bias and craving reflect some aspects of cue reactivity, but they are subserved by different mechanisms (Field and Cox, 2008). It is generally suggested that the attention bias to substance-related cues consists of early (e.g., sensory processing) and late (e.g., perceptual and cognitive processing) stages (Fehr et al., 2006; van Son et al., 2018). Moreover, substance users’ motivation for substance may bias/alter the cognitive processing of substance-related stimuli (Field and Cox, 2008). Some studies have found that the craving reduced by rTMS was associated with the improvement of cognitive function (Naish et al., 2018). One multi-center experiment conducted by our group also identified the effects of rTMS on craving and cognitive performance (Su et al., 2020a). However, whether the intervention of DLPFC affects all stages of attentional processing or merely the cognitive processing stage is currently unclear. Neurobiological results as obtained from electroencephalograph (EEG) time-domain information combined with the Addiction Stroop paradigm might contribute to the understand this problem. The Addiction Stroop paradigm for MA patients has been developed by Jiang and colleagues (Haifeng et al., 2015). In addition, a systematic review suggested that most substance-dependent patients have abnormally increased neural oscillation in the beta band, and the disrupted beta oscillation is associated with the pathological execution (Ray and Cole, 1985; Richter et al., 2017; Newson and Thiagarajan, 2018) and the response process (e.g., selection and preparation) (Davis et al., 2012; van Ede and Maris, 2013). Therefore, we followed this question in an RCT study and hypothesized that the neural oscillation in the beta band may be involved in the attention bias to substance-related stimuli and could ultimately be altered by rTMS.

In summary, in order to understand the modulation of rTMS targeting DLPFC in MA-related cues of MA patients, the aim of this article was threefold: (1) to find the difference in the attention bias and the craving between the DLPFC intermittent theta-burst stimulation (iTBS) group and the sham iTBS group after 20 treatment sessions; (2) to explore changes of ERP components in a MA cue-related attention process before and after active iTBS and sham iTBS, as well as its correlation with behavioral changes; and (3) to explore changes of EEG time-frequency components in a MA cue-related attention process before and after active iTBS and sham iTBS.

## Materials and Methods

### Study Design

Fifty-seven patients (20 females) with methamphetamine use disorder (MUD) participated in this randomized controlled study, which was part of a multi-centric clinical study (Su et al., 2020a). Patients were from two drug rehabilitation centers and both met the inclusion criteria as follows: (1) met the DSM-5 criteria for severe MUD; (2) more than 9 years of education; (3) age 18 years or older; and (4) normal vision and audition. Exclusion criteria were (1) serious physical or neurological illness, a diagnosis of any other psychiatric disorder under DSM-5 criteria (except for nicotine use disorder); and (2) any contraindications to rTMS. Baseline demographic data were investigated within the first 1–3 months after the subjects provided the signed informed consent. Patients were assigned to either the real rTMS group (*n* = 35) or the sham group (*n* = 22) (one center used 2:1 allocation methods) according to the random number table (see the CONSORT flow diagram in Supplementary Figure 1).

The study was approved by the Institutional Review Board and the Ethics Committee of Shanghai Mental Health Center and was in accordance with the principles of the Declaration of Helsinki. The protocol has been registered online at ClinicalTrials.gov (ID no. NCT02713815).

### Treatment

#### rTMS Protocol

Theta-burst stimulation (TBS) setting was used in the present study. As a new form of rTMS, the iTBS could induce the long-term potentiation by simulating the endogenous theta rhythm (Suppa et al., 2016). Four weeks of iTBS stimulation over the left DLPFC (3-pulse 50-Hz bursts given at every 200 ms, 2 s on and 8 s off for 5 min per session, 900 pulses, 100% resting motor threshold, five sessions per week) was performed in patients of the active iTBS group. The coil position was identified by using the Beam F3 method. Using the Beam F3 method to determine the stimulation location requires three scalp measurements for calculation, namely, the nasion–inion distance, the left tragus–right tragus distance through the scalp vertex, and the head circumference. The head circumference was measured at the FPz–Oz plane in the 10–20 EEG system. Based on these three measured values, the Beam F3 algorithm can provide individualized F3 positions (Beam et al., 2009; Mir-Moghtadaei et al., 2015). Transcranial magnetic stimulation (TMS) was performed using a MagPro X100 device (MagVenture, Farum, Denmark) with a figure-8-shaped MCF-B70 stimulation coil. The coil is unidirectional. The patients in the sham group received the iTBS stimulation with the same stimulation parameters while the coil rotated 180° away from the skull. Both groups could hear the sound of the stimulation pulse; however, patients of the sham iTBS group did not receive substantial magnetism across the cortex. The training researchers, who did not participate in data collection, performed the treatment for patients in separate rooms. After randomization, the motor threshold was determined as our previous study (Su et al., 2017). Patients were blind to their treatment protocol before and during treatment and were told not to uncover any treatment details with blinded raters.

#### Standardized Treatment

All patients received the regular therapy program in the rehabilitation center. The therapy program included detoxification, psychological and behavioral therapy, medical care, and anti-relapse education (Chen et al., 2019; Wu et al., 2019). In addition, the medical treatment service is provided for patients with specific needs. No medications were used throughout the duration of rTMS treatment. Standardized treatments were completed by medical staff and psychological counselors in the rehabilitation center. They are not involved in the design and setting of the present study.

### Craving Evaluation

The Visual Analog Scale (VAS) was used to assess the craving for MA use. VAS is currently the commonly used tool to assess the cravings of patients with substance use disorders and has been used in multiple studies (Carter and Tiffany, 1999; Norberg et al., 2016). Although several studies have shown that craving may be related to indicators found by EEG, skin conduction detector, and other tools (Carter and Tiffany, 1999; Norberg et al., 2016), it still cannot replace the self-reported craving of subjects. Therefore, this study chose to use VAS to assess patients’ craving. The VAS scale ranges from 0 mm (corresponding to “no craving”) to 100 mm (representing “highest craving intensity ever experienced for MA”). During the evaluation, patients watched pictures of MA-related paraphernalia (straw, tinfoil, bottle, etc.) for 5 min and filled out the scale. The same cue-related pictures were used throughout the repeated assessment. While watching the pictures, patients were asked to recall the last time they used MA. The craving was evaluated at baseline (T0), post 1 week of intervention (T1), post 2 weeks of intervention (T2), post 3 weeks of intervention (T3), and post 4 weeks of intervention (T4).

### MA Addiction Stroop Task

Attention bias to MA-related cues was assessed using the MA Addiction Stroop Task. In the field of substance use disorders, the modified Addiction Stroop paradigm is used to evaluate the attention bias of substance-dependent patients (Cox et al., 2006). The Addiction Stroop paradigm used in this study has been suggested to reflect the attentional bias of MUD (Haifeng et al., 2015). Eight MA-related target words and eight neutral words were included in this task. There was no statistically significant difference in pleasure, arousal, and familiarity between the two types of words (Jiang, 2014). Patients were informed to press one of the four keys according to the word’s color (red, yellow, green, and blue) while the patients were told not to ignore the meaning of the word. Each of the four keys was marked with a specific color to indicate the mapping between the key and the color of the word. Patients were required to press the key using the dominant hand as fast as possible. Each word remained on the screen for 3000 ms and was presented 16 times. The order of each presented word was set to be random, and the same category of the words was set not to appear three times consecutively. Fixation cross and the following word were presented on a black background 75 cm away from the eyes. Two behavioral measurements were calculated for this paradigm: (1) reaction time of each type of words was calculated for key pressing on the color-marked key in the correct trials; and (2) error rate of each type of words was calculated as the proportion of the number of errors against the total number of trials. Further details concerning this task can be found in the published works by our group (Haifeng et al., 2015). All evaluations were performed following the standardized instructions handbook by trained researchers. The MA Addiction Stroop Task with EEG recording was conducted at baseline (T0) and post 4 weeks of intervention (T4).

### EEG Data Recording and Preprocessing

While patients performed the Stroop Task, EEG data were recorded from a high-density 64-channel electrodes cap (BrainCap; Asiacut, Germany)^1^ with reference electrodes at the tip of the nose. The vertical electrooculograms (EOGs) were recorded on the up and down sides of the right eye, and the horizontal EOGs were recorded on the outer eyes of both sides. The impedances were kept below 5 kΩ with the sampling frequency at 1000 Hz. The online signal filtering was set between 0.1 and 200 Hz.

An offline bandpass filter of 0.1–30 Hz and a notch filter of 50 Hz were used. Independent component analysis (ICA) was used to correct eye movement and heartbeat artifacts. Epochs were extracted from −200 to 1000 ms relative to the onset of the word, and averaged EEG data from −200 to 0 ms were applied for baseline correction. Then, all EEG epochs were processed for artifact detection by visual inspection and EEGLAB, including the examination for peak-to-peak deflection exceeding ± 100 mV threshold and the detection of obvious eye movement/blinks. The epoch with a maximal 150-mV threshold amplitude difference within a 200-ms width and 50-ms step moving windows was also discarded. In order to ensure the quality of data, patients with more than 20% (10/50) of bad epochs for each condition and/or five bad channels were excluded from the analysis. All preprocessing of the EEG data was conducted by EEGLAB (Version: 12.0.2.6b)^2^ based on MATLAB (Version: 2013b)^3^.

### Event-Related Potentials (ERPs)

**FIGURE 1:**
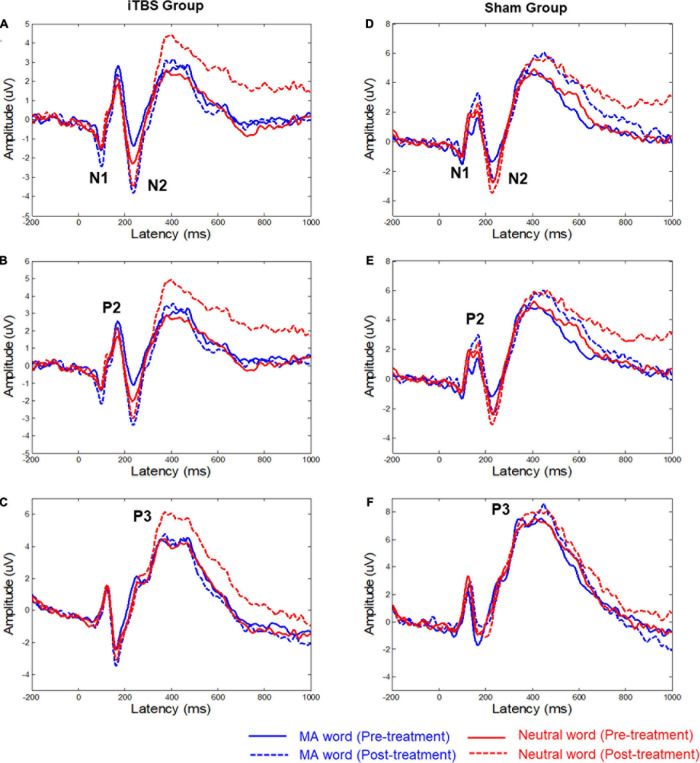
Grand mean averages (μV) of event-related potentials to the two types of words in the two groups. **(A,D)** The average ERP waveforms of six electrodes (FZ/F1/F2/FCZ/FC1/FC2), the time-by-group effect was significant (*p* = 0.04) on N1 Amplitude^*MA*−*Neutral*^. **(B,E)** The average ERP waveforms of six electrodes (FZ/F3/F4/FCZ/FC3/FC4). **(C,F)** The average ERP waveforms of six electrodes (CPZ/CP3/CP4/PZ/P3/P4). The significant time-by-group effect was identified based on P3 latency^*MA*−*Neutral*^ (*p* = 0.02). MA, methamphetamine.

According to the brain EEG topography (Supplementary Figures 2, 3) and grand average of ERP amplitudes (Figure 1), four main components were analyzed: N1, N2, P2, and P3. N1 and N2 were mainly distributed in the middle frontal area. Hence, statistical analysis was conducted based on the latency and the amplitude of N1 and N2 obtained from the six electrodes in the frontal area: FZ/F1/F2/FCZ/FC1/FC2 (Figure 1). P2 was mainly distributed in the frontal area, so the statistical analysis was focused on the six frontal electrodes: FZ/F3/F4/FCZ/FC3/FC4. For P3, the statistical analysis was focused on the six parietal electrodes: CPZ/CP3/CP4/PZ/P3/P4. For each patient, the amplitude of each component was obtained by averaging the amplitudes in the 50-ms time range that centered at the group peak amplitude (see Supplementary Figures 2, 3 for the specific time ranges). The latency of each component was determined by the peak latency.

### Time-Frequency Analysis

Time-frequency analysis was conducted on the preprocessed EEG time course ranging from −500 to 1000 ms relative to the onset of the word. In the current study, the time-frequency analysis was conducted to show which frequency component was involved in the attentional bias more than the fine temporal characteristics of the frequency component during the task. The potential frequency band to be tested in present study lies in the range of 5–30 Hz (e.g., theta, alpha, and beta), which was well documented in previous studies that concern attention and cognitive control (Klimesch, 2012; Clayton et al., 2015). The short-time Fourier transform (STFT) method could provide a sufficiently high-frequency resolution to cover this frequency range and achieves a good trade-off between time resolution and frequency resolution. Moreover, the STFT method is better at avoiding the contamination of the estimates of post-stimulus EEG responses by pre-stimulus activity (Zhang et al., 2012). Therefore, the time-frequency distribution of the EEG time course was obtained using a STFT with a fixed 200-ms Hanning window. For each experimental condition of each participant, we estimated spectral power at frequencies between 1 and 30 Hz in 0.5-Hz steps. Event-related power changes were calculated as the percentage change of power relative to the baseline (−200 to 0 ms).

### Statistical Analyses

Student’s *t*-tests were used to test the difference in the continuous variable between the two groups, and chi-square tests were used for comparison of dichotomous variables. If the assumption of normality and/or homogeneity of variance was violated, non-parametric tests were used.

For each ERP component, the difference in amplitude between drug-related words and neutral words (ERP^MA–Neutral^), which reflect patients’ attention bias to drug-related cues, was used to evaluate the intervention effect between the two groups. Repeated-measures analyses of variance with intergroup variable (active TMS vs. sham TMS) × intragroup variable (time) were performed to evaluate the main effect of time, group, and group–time interaction on the outcomes, respectively, and the *p*-value was corrected by Greenhouse–Geisser correction when necessary. For the significant time-by-group effect, *post hoc* analysis was conducted and the Bonferroni correction was used for multiple comparison tests. The Pearson correlation between changes of clinical performance (i.e., craving) and changes of significant ERP components in the two groups were also conducted, as the Bonferroni correction was used for multiple correlation tests.

Considering that the intervention in different groups might cause changes in different time-frequency domains, data-driven statistical testings on time-frequency maps were separately performed for the two groups. For each group, we calculated two contrasts based on time series of power changes: (1) “MA-related word > Neutral word” before the intervention (MNT1) and (2) “MA-related word > Neutral word” after the intervention (MNT2). These two contrasts were calculated separately for each EEG channel. To investigate the spectral changes by intervention, dependent-sample *t*-tests were conducted on “MNT2 > MNT1” collapsed over all channels. A cluster-based permutation test was further conducted to correct multiple comparisons. The number of random permutations using the Monte Carlo method was set to 5,000, and adjacent points exceeding *p* < 0.05 were identified as a significant cluster. All data were analyzed with SPSS 22 and MATLAB 2013b.

## Results

### Demographics and MA Use History

Eight individuals (including five patients in the active group and three in the sham group) with more than five bad channels and/or more than 20% (10/50) of bad epochs for each condition were excluded from the subsequent analysis. There were no significant differences between the active (*n* = 30) and sham iTBS (*n* = 19) group in terms of age, gender, or years of education (Table 1). In aspects of MA use history, no difference between the two groups was found in the age of first substance use, total years of substance use, and abstinent times.

**TABLE 1:** Baseline demographic of methamphetamine-dependent patients in two treatment groups.

	**Active iTBS group (*n* = 30)**	**Sham iTBS group (*n* = 19)**	***χ*^2^/*F***	***p*-value**
Age (SD)	29.66 (4.70)	30.73 (6.68)	–0.66	0.51
Gender (F/M)	10/20	10/11	1.06	0.30
Years of education (SD)	8.69 (2.39)	9.05 (2.36)	–0.52	0.61
Age of first substance use (SD)	23.37 (5.15)	23.82 (7.53)	–0.25	0.80
Total years of substance use (SD)	5.00 (3.21)	5.00 (3.89)	0.00	1.00
Abstinent times (months) (SD)	2.63 (1.42)	3.05 (1.40)	–1.01	0.32
Baseline craving (VAS) (SD)	62.29 (31.43)	50.41 (29.76)	1.32	0.19

*iTBS, intermittent theta-burst stimulation; VAS, visual analog scale.*

### Craving and Behavioral Data Results

After intervention, significant time effect (*F* = 20.60, *p* < 0.01), group effect (*F* = 5.16, *p* = 0.03), and time-by-group effect (*F* = 29.24, *p* < 0.01) on craving scores were indicated (Table 2). In addition, a significant time-by-group effect (*F* = 4.24, *p* = 0.04) on the error rate of MA words was demonstrated. No significant time effect (*F* = 0.06, *p* = 0.81) and group effect (*F* = 0.07, *p* = 0.79) were found on the error rate of MA words. There is also a significant time effect (*F* = 4.11, *p* = 0.05) on the error rate of neutral trials, while no group effect (*F* = 1.03, *p* = 0.32) and time-by-group effect (*F* = 2.94, *p* = 0.09) were found.

**TABLE 2:** Craving and Addiction Stroop Task performance of patients after DLPFC iTBS intervention.

	**Active iTBS group (*n* = 30)**	**Sham iTBS group (*n* = 19)**	**Effect**					
			**Time**		**Group**		**Interaction**	
			***F***	***p*-value**	***F***	***p*-value**	***F***	***p*-value**
**Self-report craving**								
Baseline (T0)	62.29 (31.43)	50.41 (29.76)	20.60	<0.01**	5.16	0.03*	29.24	<0.01**
Post 1st week of intervention (T1)	37.74 (26.48)	33.05 (30.23)						
Post 2nd week of intervention (T2)	33.43 (24.99)	52.82 (29.83)						
Post 3rd week of intervention (T3)	24.74 (22.63)	54.95 (26.08)						
Post 4th week of intervention (T4)	14.46 (15.72)	52.18 (28.00)						
**Addiction Stroop Task**								
ΔRT^MA–Neutral^ (ms) (SD) (T0)	11.12 (28.51)	10.01 (29.10)	1.51	0.23	0.76	0.39	0.00	0.98
ΔRT^MA–Neutral^ (ms) (SD) (T4)	3.32 (18.97)	0.74 (24.40)						
Error rate of MA words (SD) (T0)	0.10 (0.11)	0.05 (0.03)	0.06	0.81	0.07	0.79	4.24	0.04*
Error rate of MA words (SD) (T4)	0.05 (0.04)	0.07 (0.09)						
Error rate of neutral trials (SD) (T0)	0.09 (0.10)	0.04 (0.03)	4.11	0.05*	1.03	0.32	2.94	0.09
Error rate of neutral trials (SD) (T4)	0.04 (0.05)	0.06 (0.09)						

^*MA–Neutral*^The reaction time in MA condition minus that in neutral condition. T0, baseline; T1, post 1 week of intervention; T2, post 2 weeks of intervention; T3, post 3 weeks of intervention; T4, post 4 weeks of intervention; iTBS, intermittent theta-burst stimulation; DLPFC, dorsolateral prefrontal cortex; rTMS, repetitive transcranial magnetic stimulation; MA, methamphetamine; RT, reaction time.

**p < 0.01; *p < 0.05.

### ERP Results

Figure 1 shows the ERP waveforms for the two conditions (i.e., MA word and neutral word) with associated EEG electrode sites. Supplementary Table 1 shows the difference in mean amplitude and latency of the ERP components (i.e., N1, P2, N2, and P3) between MA word and neutral word (before and after treatment).

For the N1 component, the results showed that the time-by-group effect was significant (*F* = 4.43, *p* = 0.04) on N1 Amplitude^MA–Neutral^ (Figure 1 and Supplementary Table 1). *Post hoc* analysis showed no significant change in N1 Amplitude^MA–Neutral^ in both active iTBS group (*t* = 1.50, corrected *p* = 0.284) and sham group (*t* = −1.49, corrected *p* = 0.29). No significant effects on the latency of N1 were observed.

For the P3 component, the significant time-by-group effect was identified based on P3 latency^MA–Neutral^ (*F* = 5.67, *p* = 0.02). *Post hoc* analysis showed no significant change in P3 latency^MA–Neutral^ in both active iTBS group (*t* = 1.61, corrected *p* = 0.22) and sham group (*t* = −1.76, *p* = 0.17). No significant effects on the amplitude of P3 were observed.

There was no significant effect for N2 and P2 components.

### Correlation Between ERP Components and Craving

In the active group, further correlation analyses illustrate that changes of N1 Amplitude^MA–Neutral^ were positively correlated with cue-induced craving (*r* = 0.48, corrected *p* = 0.03). There was no significant correlation between changes of P3 latency^MA–Neutral^ with craving (*r* = −0.13, *p* = 0.49).

In the sham group, further correlation analyses demonstrated no significant correlation between changes of craving with P3 latency^MA–Neutral^ (*r* = −0.32, *p* = 0.18) and changes of N1 Amplitude^MA–Neutral^ (*r* = 0.34, *p* = 0.16).

### Time-Frequency Results

**FIGURE 2:**
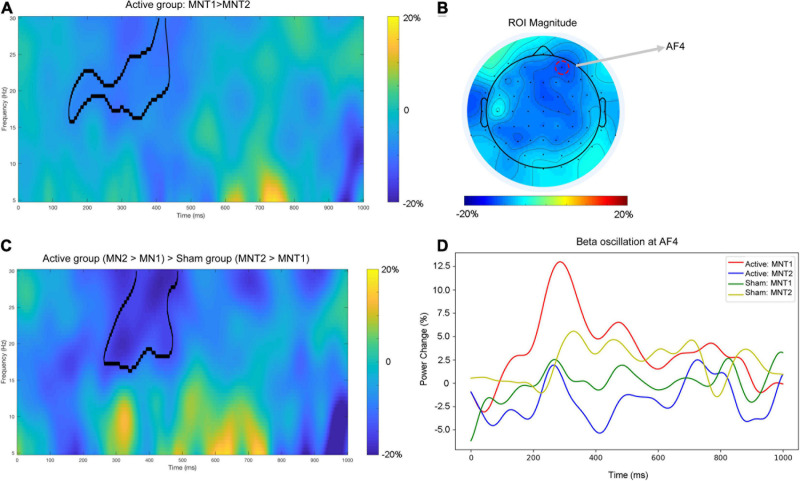
**(A)** The time-frequency map averaged over all participants for the contrast ‘MNT2 > MNT1’ for Active group. The area framed by black line indicates the significant cluster. **(B)** The topographical distribution of power change in beta band (averaged across the significant cluster in **A)**. The red dashed circle indicating the electrode shows the strongest power change (i.e., AF4). **(C)** The time—frequency map average over all participants for the interaction contrast ‘Active (MNT2 > MNT1) > Sham (MNT2 > MNT1)’. The area framed by the black line indicates the significant cluster. **(D)** The time courses of power changes in beta-band extracted from AF4. MNT1, ‘MA-related word > Neutral word’ before intervention; MNT2, ‘MA-related word > Neutral word’ after intervention.

For the active group, a cluster at beta band (16–30 Hz) showed significantly reduced power (desynchronization) after the intervention (Figure 2A). The strongest power reduction was localized at the electrode AF4 (Figure 2B). No significant cluster was observed for the sham group. The presence of beta desynchronization in the active group versus the absence of beta desynchronization in the sham group was confirmed by a further interaction contrast “Active (MNT2 > MNT1) > Sham (MNT2 > MNT1)” (Figure 2C). Time courses of power changes in beta band were also extracted from an example electrode (AF4) and shown in Figure 2D.

## Discussion

This study focused on the role of iTBS in modulating the attention bias of MA-related cues in MA patients. At the behavioral level, the active iTBS treatment significantly reduced the error rate of MA words. At the neural level, there was a significant time-by-group effect for the N1 amplitude^MA–Neutral^ and P3 latency^MA–Neutral^ components. In addition, time-frequency analysis suggested that the active iTBS treatment significantly reduced the beta band power, especially on electrodes in the frontal central area. Taken together, these results indicated that iTBS applied to left DLPFC may influence brain electrophysiological changes during the attentional processing of MA words in MA patients. The potential mechanism could be hypothesized that iTBS reduced the attentional bias to drug-related information in MA patients.

### The Effect of ITBS on Early ERP Components of MA Addiction Stroop

The amplitude of early ERP components (e.g., N1 and P2) has been generally considered as reflecting the arousal level to the exterior stimulus (e.g., visual materials or auditory materials) (Field and Cox, 2008). In the present research, we compared the ERP amplitude differences between MA words and neutral words, which quantify the arousal level induced by MA-related cues relative to neutral cues (i.e., abnormal hyperarousal to MA-related cues). Previous studies implied that drug users (e.g., heroin and cannabis) had significantly larger N1 amplitude compared to healthy controls (Zhao et al., 2017; Ruglass et al., 2019). The significant time-by-group effect was identified for the N1 amplitude^MA–Neutral^. However, *post hoc* analysis found that there was no significant decrease in N1 amplitude^MA–Neutral^ in the active iTBS group, suggesting that active iTBS stimulation did not have more effect on the early information processing of MA-related cues. Interestingly, Zikopoulos and Squire’s studies suggested that DLPFC plays an important role in the function of sensory processing (Zikopoulos and Barbas, 2007; Squire et al., 2013). Recently, Zhang L. et al. (2018) found that high-frequency rTMS on the DLPFC could restore attention bias to negative emotional information in MA patients. Besides, no significant iTBS treatment effect on N2 component was found, suggesting that iTBS targeting the DLPFC has a selective effect on early ERP components. Besides, the previous study believes that different forms of stimulus (e.g., text and pictures) may affect the Stroop results and limit the comparability between different tasks (Jiang, 2014). Therefore, a potential interpretation is that iTBS does not have a regulatory effect on the N2 component caused by lexical stimulation, but its effect on other types of stimuli needs further exploration. However, the present study identified the positive correlation between changes in N1 amplitude^MA–Neutral^ and changes of cue-induced craving among individuals in the active group. Specifically, individuals who had a stronger decrease in N1 amplitude^MA–Neutral^ showed more reduced cravings after the intervention. Collectively, the early information processing may be involved in the generation of cue-induced craving. Therefore, our current study may provide a new perspective, which is to reduce the patient’s craving by reducing the level of early arousal to substance-related cues. However, whether the DLPFC iTBS strategy is effective on MA-related cue processing may require further studies to confirm.

### The Effect of ITBS on Late ERP Components of MA Addiction Stroop

Late ERP components reflect the process of attentional selection and executive resources needed to control the prepotent response in reaction to an exterior stimulus such as a drug-related cue (Coull, 1998; Barcelo and Cooper, 2018; Camfield et al., 2018). The significant time-by-group effect was identified for the P3 latency^MA–Neutral^. However, *post hoc* analysis found no significant decrease in P3 latency^MA–Neutral^ in the active iTBS group. Prolonged P3 latency and low P3 amplitude were suggested to be correlated with poor cognitive performance in previous studies (Ogawa et al., 2009; Euser et al., 2012). Therefore, the decrease of P3 latency^MA–Neutral^ may indicate the enhancement of patients’ cognitive control on the dominant response to MA-related cues (Del Felice et al., 2016). Interestingly, a previous study had found that the P3 latency is negatively correlated with the efficiency of dopamine D2/D3 receptors (Pogarell et al., 2011), which may mean that the shortened P3 latency implies an increase in the effectiveness of dopamine receptors. However, there is still insufficient evidence to conclude whether TMS can affect the dopamine level and dopamine receptor function of the patients with substance dependence (Sanna et al., 2020). The present study did not show the effect of active DLPFC iTBS treatment on P300 components. Therefore, the results of the present study suggest that the dopamine system may not be the main neurotransmitter system affected in the DLPFC iTBS intervention protocol. Collectively, the ERP results may not be enough to suggest the modulation effect of active iTBS treatment on early and late stages of processing of MA-related cues. Further investigation is still necessary. Actually, a previous study has found that the limbic circuit is directly related to cue reactivity (Courtney et al., 2016), and the therapeutic effect of DLPFC stimulation may be a mediating effect on the limbic circuit (Hayashi et al., 2013). Therefore, a direct intervention targeting the limbic circuit may be a potential intervention protocol for substance cue-induced attention bias and craving. Our group reported that the combined stimulation of DLPFC and ventromedial prefrontal cortex (core brain area of the limbic circuit) had a stronger craving reduction effect than simply stimulating DLPFC, which partially validated this theoretical hypothesis (Chen et al., 2020).

### The Effect of ITBS on Beta Band Power of MA Addiction Stroop

As illustrated in Figure 2, reduced power of neural oscillation (desynchronization) in the beta band was observed in active iTBS after the intervention, and this desynchronization was manifested from the right frontal to the central area of the brain. Previous studies have revealed that the neural oscillation in the beta band was involved in tasks where effortful inhibitory control was required (Lalo et al., 2008; Swann et al., 2009). A review proposed that pathological enhancement of beta band neural activity reflects the deterioration of cognitive control and behavioral flexibility (Engel and Fries, 2010). From this perspective, the reduction in beta activity in the active iTBS group might suggest improved inhibitory control on the response to MA-related cues, and this improvement might originate from the improved function of the stimulated region (i.e., DLPFC), which has also been shown to be related to response inhibition (Huang et al., 2004; Hwang et al., 2010). Moreover, the beta band activity could be related to increased GABA-mediated inhibition (Premoli et al., 2017). For instance, previous studies have shown that trials requiring less inhibitory control were associated with more GABA-mediated inhibition as well as beta power decrease when compared with those trials applying more inhibitory control (Kuhn et al., 2004; van Wouwe et al., 2016). Preclinical and human studies also demonstrate that beta band oscillations are the accumulated output of neural cells aligned by GABAergic interneuron rhythmicity (Jensen et al., 2005; Yamawaki et al., 2008). In our recent study, we found that the GABA concentration in DLPFC was lower in MA patients than in healthy controls (Su et al., 2020b). After DLPFC rTMS treatment, GABA concentration in the DLPFC increased in the patients with depression (Levitt et al., 2019), and this significant enhancement only appeared in patients who responded to treatment. Another study also suggested that the iTBS treatment increased the N100 amplitude of the TMS-evoked potential (Harrington and Hammond-Tooke, 2015), which is a marker of intracortical GABAB-mediated inhibition (Premoli et al., 2014). Taken together, the reduced beta activity by the iTBS targeted at DLPFC thus provides a potential electrophysiological marker that could inform the molecular level (e.g., GABA) to help evaluate the intervention effect on MA patients. However, further research is needed before drawing the final conclusion.

Some limitations should be mentioned. Firstly, we did not measure the follow-up performance of the Addiction Stroop Task. Tracking the fluctuation of electrophysiological components after the treatment would help elucidate the association of rTMS effect, attention bias, and patients’ clinical outcomes. In future research, it will be important to explore the long-term impact of iTBS on modulating substance-related attention bias and the progression in MUD. Secondly, although EEG has a high temporal resolution in revealing brain activity, the poor spatial resolution makes it hard to capture changes from a specific brain region and/or functional networks in accordance with the intervention. In the future, combining magnetic resonance imaging (MRI) and/or magnetoencephalography (MEG) technology will help to further clarify the specific role of rTMS on detailed neural circuits related to attention bias. Thirdly, this study clarified the cumulative modulation effect of iTBS stimulation from 20 sessions. However, the transient modulation of rTMS, the relationship between early and late components, and the optimal stimulation time point conducted to the Addiction Stroop Task are still not clear. Future studies into the above question could help improve understanding of the basic dimensions underlying precise intervention targeting cue reactivity, and ultimately lead to improving treatment. That is another important direction we are heading to.

In conclusion, to the best of our knowledge, this is the first study to investigate the effects of iTBS on substance-related attention bias by linking EEG with behavioral data. This study suggested that DLPFC iTBS has a specific potential effect on substance-related attention bias, but it does not affect the whole process of attention. These results might advance the understanding of the mechanisms in rTMS treatment for substance dependence and might assist in structuring the personalization of rTMS intervention. In addition, this study also found that craving is related to the early stage EEG components of substance-related attention processing, which may provide the effective biological marker of craving.

## Data Availability Statement

The raw data supporting the conclusions of this article will be made available by the authors, without undue reservation.

## Ethics Statement

The studies involving human participants were reviewed and approved by the Institutional Review Board and the Ethics Committee of Shanghai Mental Health Center. The patients/participants provided their written informed consent to participate in this study.

## Author Contributions

TC, HS, XL, QW, YM, CD, and WSi performed the research. TC, LW, NZ, and HJ analyzed the data. MZ, TC, and HS designed the research. TC drafted the manuscript. CZ, DX, and WSo provided the technical support. All authors have contributed to the interpretation of data, critically revised the manuscript, and approved the final version of the manuscript.

## Conflict of Interest

The authors declare that the research was conducted in the absence of any commercial or financial relationships that could be construed as a potential conflict of interest.

## Publisher’s Note

All claims expressed in this article are solely those of the authors and do not necessarily represent those of their affiliated organizations, or those of the publisher, the editors and the reviewers. Any product that may be evaluated in this article, or claim that may be made by its manufacturer, is not guaranteed or endorsed by the publisher.

## Footnotes

1
https://www.brainproducts.com/

2
https://sccn.ucsd.edu/eeglab/index.php

3
https://ww2.mathworks.cn/en/
