# Supplementary material for: Modulation of Methamphetamine-Related Attention Bias by Intermittent Theta-Burst Stimulation on Left Dorsolateral Prefrontal Cortex
Source: Front Cell Dev Biol. 2021 Aug 3;9:667476. doi: 10.3389/fcell.2021.667476 (PMC8370756; doi:10.3389/fcell.2021.667476)
Supplement: Supplementary file 1 [file Data_Sheet_1.DOCX]

**Supplementary material**

Title: Modulation of methamphetamine-related attention bias by intermittent theta-burst stimulation on left dorsolateral prefrontal cortex


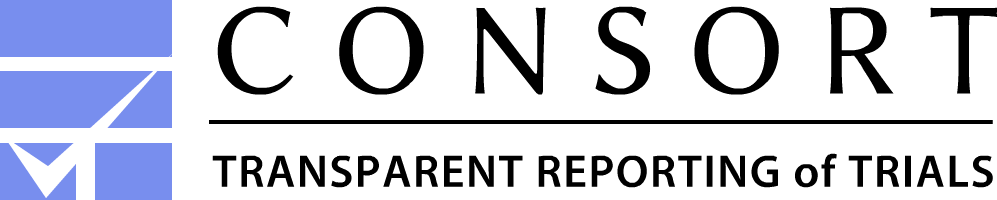


**CONSORT 2010 Flow Diagram**

## Enrollment

Assessed for eligibility (n=86)

Excluded (n=29)

♦  Not meeting inclusion criteria (n=15)

♦  Declined to participate (n=11)

♦  Other reasons (n=3)

Randomized (n=57)

## Allocation

Allocated to sham iTBS (n=22)

Allocated to active iTBS (n=35)

Excluded (n=8)

♦  The quality of EEG data did not meet the criteria(n=8)

## Analysis

Analysed (n=30)

Analysed (n=19)

Figure S1. CONSORT 2010 flow diagram.

Figure S2. The brain topography of ERP component in condition of MA words. (A) The brain topography of N1 component. (B) The brain topography of P2 component. (C) The brain topography of N2 component. (D) The brain topography of P3 component.

Figure S3. The brain topography of ERP component in condition of Neutral words. (A) The brain topography of N1 component. (B) The brain topography of P2 component. (C) The brain topography of N2 component. (D) The brain topography of P3 component.

| Table S1. ERP changes of Addiction Stroop Task in patients after DLPFC iTBS treatment. | | | | | | | | | | | | | |
| --- | --- | --- | --- | --- | --- | --- | --- | --- | --- | --- | --- | --- | --- |
|  | Active iTBS group  (n=30) | Sham iTBS group  (n=19) | | Effect | | | | | | | | | |
|  |  |  |  | Time | | |  | Group | |  | Interaction | |  |
|  |  |  |  | F | | P value |  | F | P value |  | F | P value |  |
| △N1 Lat ^MA-Neutral^ (SD) (T0) | -0.21 (12.06) | -1.32 (16.92) | 0.42 | | 0.52 | |  | 0.02 | 0.89 |  | 0.17 | 0.68 |  |
| △N1 Lat ^MA-Neutral^ (SD) (T4) | 1.69 (12.30) | -0.11 (17.69) |  |  |  |  |  |  |  |  |  |  |  |
| △N1 Amp^MA-Neutral^ (SD) (T0) | 0.06 (2.14) | -0.50 (1.18) | 0.05 | | 0.83 | |  | 0.04 | 0.84 |  | 4.43 | **0.04*** |  |
| △N1 Amp ^MA-Neutral^ (SD) (T4) | -0.52 (1.37) | 0.20 (1.40) |  |  |  |  |  |  |  |  |  |  |  |
| △P2 Lat ^MA-Neutral^ (SD) (T0) | -0.17 (13.03) | 6.79 (14.64) | 0.54 | | 0.46 | |  | 0.93 | 0.34 |  | 2.50 | 0.12 |  |
| △P2 Lat ^MA-Neutral^ (SD) (T4) | 1.93 (9.92) | 1.00 (17.56) |  |  |  |  |  |  |  |  |  |  |  |
| △P2 Amp ^MA-Neutral^ (SD) (T0) | 0.90 (3.05) | 0.18 (1.19) | 0.68 | | 0.41 | |  | 0.06 | 0.81 |  | 3.19 | 0.08 |  |
| △P2 Amp ^MA-Neutral^ (SD) (T4) | 0.04 (2.11) | 0.49 (1.75) |  |  |  |  |  |  |  |  |  |  |  |
| △N2 Lat ^MA-Neutral^ (SD) (T0) | 0.35 (10.34) | 0.95 (6.33) | 0.24 | | 0.63 | |  | 0.01 | 0.94 |  | 0.12 | 0.73 |  |
| △N2 Lat ^MA-Neutral^ (SD) (T4) | 2.24 (12.08) | 1.26 (15.51) |  |  |  |  |  |  |  |  |  |  |  |
| △N2 Amp ^MA-Neutral^ (SD) (T0) | 0.62 (1.92) | 0.94 (2.03) | 3.74 | | 0.06 | |  | 0.01 | 0.96 |  | 0.93 | 0.34 |  |
| △N2 Amp ^MA-Neutral^ (SD) (T4) | -0.23 (1.49) | 0.13 (2.18) |  |  |  |  |  |  |  |  |  |  |  |
| △P3 Lat ^MA-Neutral^ (SD) (T0) | 4.66 (16.40) | -5.47 (19.39) | 0.12 | | 0.73 | |  | 0.02 | 0.90 |  | 5.67 | **0.02*** |  |
| △P3 Lat ^MA-Neutral^ (SD) (T4) | -4.38 (20.04) | 6.68 (22.09) |  |  |  |  |  |  |  |  |  |  |  |
| △P3 Amp ^MA-Neutral^ (SD) (T0) | 0.20 (2.07) | 0.27 (1.74) | 1.69 | | 0.20 | |  | 0.01 | 0.97 |  | 0.02 | 0.89 |  |
| △P3 Amp ^MA-Neutral^ (SD) (T4) | -0.15 (2.12) | -0.18 (2.16) |  |  |  |  |  |  |  |  |  |  |  |
| ^MA-Neutral^The EEG variable in MA condition minus that in neutral condition. N1 and N2 component were analyzed at six electrodes of frontal area (FZ/F1/F2/FCZ/FC1/FC2), P2 component were analyzed at six electrodes of frontal area (FZ/F3/F4/FCZ/FC3/FC4), P3 component were analyzed at six electrodes of parietal area (CPZ/CP3/CP4/PZ/P3/P4).  **p˂0.01; *p˂0.05. Amp=amplitude; Lat=latency. T0=baseline; T4 = post 4 week of intervention. | | | | | | | | | | | | | |
